# Omega-3 polyunsaturated fatty acids in diabetic-associated cognitive dysfunction: a nutritional therapeutic perspective

**DOI:** 10.3389/fnut.2025.1651304

**Published:** 2025-08-29

**Authors:** Chunying Cui, Yan Yang, Pengfei Liu, Yan Gao, Daqing Song, Shangbin Li

**Affiliations:** ^1^Emergency Department, Jining No.1 People's Hospital, Jining, Shandong, China; ^2^Department of Geriatric Neurology, Shandong Provincial Hospital Affiliated to Shandong First Medical University, Jinan, Shandong, China; ^3^Department of Cardiology, Jining No.1 People's Hospital, Jining, Shandong, China; ^4^Institute of Emergency and Critical Care of Jining Medical Research Academy, Jining, Shandong, China; ^5^Department of Geriatrics, Shandong Provincial Hospital Affiliated to Shandong First Medical University, Jinan, Shandong, China

**Keywords:** omega-3 polyunsaturated fatty acids, diabetic-associated cognitive dysfunction, insulin resistance, neuroinflammation, synaptic plasticity, gut-brain axis

## 1 Introduction

The intersection between metabolic dysfunction and cognitive impairment represents one of the most pressing yet under-addressed challenges in modern medicine. With the global prevalence of diabetes reaching epidemic proportions, currently affecting more than 800 million adults worldwide, the neurological sequelae of this condition—particularly diabetes-associated cognitive dysfunction (DACD)—have emerged as a critical public health concern requiring immediate attention (1). DACD manifests through progressive memory impairment, executive function decline, and accelerated neurodegenerative processes (2, 3). These neurological deficits significantly impair quality of life while simultaneously exacerbating diabetes self-management challenges, thereby establishing a detrimental feedback loop between metabolic dysregulation and cognitive deterioration. Although the mechanisms underlying cognitive dysfunction in patients with diabetes are currently unclear, emerging evidence suggests that the interplay of insulin resistance (IR), chronic neuroinflammation, synaptic dysfunction, and gut-brain axis dysregulation are pivotal drivers of DACD pathogenesis (4–6). Contemporary therapeutic strategies for DACD demonstrate limited efficacy, predominantly emphasizing glycemic management while inadequately addressing the complex pathophysiology of diabetes-induced neural injury. This therapeutic gap highlights the critical need for interventions that simultaneously target the metabolic, inflammatory, and neurodegenerative components of DACD. Accumulating evidence suggests that omega-3 polyunsaturated fatty acids (PUFAs), specifically eicosapentaenoic acid (EPA) and docosahexaenoic acid (DHA), may represent promising therapeutic candidates for DACD. Table 1 synthesizes preclinical and clinical evidence documenting the pleiotropic neuroprotective mechanisms of omega-3 PUFAs, encompassing therapeutic ingredients, targets of action, and research results.

**Table 1:** Neuroprotective and metabolic benefits of omega-3 PUFAs: evidence from preclinical and clinical studies.

**Category**	**Model/Population**	**Intervention**	**Targeted pathway**	**Primary outcomes**	**Reference**
Preclinical studies	Diabetic rodent models	DHA supplementation	IRS/PI3K/AKT signaling	Improved hippocampal insulin sensitivity, reduced amyloid/tau pathology, rescued spatial memory	(18)
	Diabetic mice	HFD containing 0.5% EPA	P62/KEAP1/NRF2 antioxidant pathway	Reduced oxidative stress, preserved synaptic plasticity	(35)
	Neuroblastoma cells	EPA-rich crude extracts	BDNF-CREB signaling	Increased synaptogenesis, enhanced neurite outgrowth	(41)
	Transgenic fat-1 mice	Omega-3 PUFA mix	Gut-brain axis	Anti-inflammatory, microbiota-modulating, strengthened intestinal/BBB barriers	(47)
	C57BL/6JNifdc mice	Soybean oil + lard (omega-3 PUFAs mix)	SCFA/ERK/CREB/BDNF pathway	Reversed cognitive impairment, normalized hippocampal fatty acid composition	(9)
	PM2.5-exposed young rats	DHA (100 mg/kg/day)	CREB/BDNF signaling pathway	Improving synaptic plasticity and cognitive impairment	(39)
Clinical study	T2DM patients	EPA+DHA (3:2 ratio)	SIRT1 activation	Enhanced mitochondrial biogenesis, improved glycemic control	(20)

HFD, high-fat die.

Compelling preclinical evidence supports omega-3 PUFAs as biologically rational candidates for DACD management, based on their indispensable roles in both the structural architecture of neural tissue (particularly DHA's enrichment in synaptic membranes) and functional regulation of neuroprotective pathways. DHA accounts for ~30% of total brain phospholipid content, reflecting its fundamental role in preserving neuronal membrane stability and synaptic functionality. Beyond these structural contributions, omega-3 PUFAs exhibit significant biological activity in regulating neural insulin sensitivity, attenuating neuroinflammatory processes, and promoting neuroplastic adaptations—each representing key pathophysiological mechanisms in DACD (7–10). Emerging research has increasingly implicated the gut-brain axis in the pathogenesis of DACD, uncovering novel therapeutic opportunities for omega-3 PUFAs. Experimental evidence demonstrates that these bioactive lipids can modulate intestinal microbial ecology by promoting commensal taxa (e.g., Bifidobacterium, Lactobacillus) and suppressing pathobionts (11, 12). Such microbiota alterations may elevate circulating levels of neuroactive microbial metabolites, particularly short-chain fatty acids, thereby establishing a systemic milieu conducive to neural homeostasis.

Despite robust preclinical evidence, clinical outcomes of omega-3 PUFA supplementation in DACD remain heterogeneous. While certain randomized controlled trials report significant cognitive improvements in diabetic cohorts, others demonstrate only marginal benefits. The observed heterogeneity in clinical outcomes likely stems from variations in study methodologies, including differences in design parameters and population characteristics. Furthermore, growing evidence indicates that genetic factors, particularly APOE polymorphisms, may serve as key determinants of interindividual responsiveness to omega-3 PUFAs interventions. This is because the APOE ε4 genotype alters DHA metabolism when supplemented with omega-3 PUFAs, resulting in a lower plasma response to omega-3 PUFAs in APOE ε4-positive than in APOE ε4-negative individuals. This study systematically evaluated the multi-target mechanism of action of omega-3 PUFAs in DACD and identified key knowledge gaps for future translational research.

## 2 Omega-3 PUFAs: metabolic maestros in DACD management

Accumulating evidence indicates that IR impairs cerebral insulin signaling, resulting in deficient neuronal glucose uptake and metabolic dysfunction. Central insulin resistance (CIR) represents a defining pathological feature of diabetes that promotes cognitive dysfunction through multifaceted mechanisms including impaired cerebral glucose metabolism, compromised synaptic plasticity, disrupted neurotrophic signaling, and dysregulated energy homeostasis (13, 14). The dysregulation of the insulin receptor substrate/phosphatidylinositol 3-kinase/protein kinase B (IRS/PI3K/AKT) pathway is a direct trigger of IR (15). Contemporary research reveals omega-3 PUFAs, particularly EPA and DHA, as pleiotropic modulators of the metabolic perturbations underlying DACD. Omega-3 PUFAs ameliorate CIR through three complementary mechanisms: (i) modulation of membrane fluidity in neuronal organelles (endoplasmic reticulum and Golgi apparatus), (ii) activation of peroxisome proliferator-activated receptor gamma (PPARγ) receptors with concomitant suppression of endoplasmic reticulum stress, and (iii) restoration of IRS/PI3K/AKT signaling pathways (8, 16, 17). In the experimental rodent model, DHA supplementation restored systemic glucose homeostasis, ameliorated hippocampal insulin sensitivity, and reduced hippocampal amyloid formation and tau phosphorylation, while rescuing hippocampus-dependent spatial memory and cognitive deficits (18). Sirtuin1 (SIRT1) is a conservative nicotinamide adenine dinucleotide (NAD)^+^-dependent deacetylase that is mainly located in the nucleus, which closely correlates with mitochondrial biogenesis, lipid metabolism, and metabolic fluxes (19). Available evidence suggests that omega-3 PUFAs enhance mitochondrial biogenesis and oxidative metabolism by upregulating SIRT1 expression, which in turn counteracts diabetes-induced cerebral hypometabolism (20, 21). The pleiotropic actions of omega-3 PUFAs extend beyond metabolic regulation to encompass suppression of microglia-dependent neuroinflammation and oxidative stress, addressing multiple pathological cascades in parallel.

## 3 Omega-3 PUFAs: simultaneously targeting neuroinflammatory and oxidative pathways

Emerging research reveals a complex interplay between CIR and neuroinflammation in DACD. Chronic hyperglycemia fundamentally alters microglial functional phenotypes, converting these central nervous system immune sentinels from their resting surveillance state to damaging M1-like inflammatory effectors. This polarization is characterized by increased secretion of damaging cytokines (IL-1β, TNF-α) and activation of nuclear factor kappa-B (NF-κB) signaling and the NOD like receptor family pyrin domain containing 3 (NLRP3) inflammasome, leading to elevated levels of IL-6 and IL-18 (22, 23). The diabetic milieu fosters a deleterious neuroimmune cycle where microglia-mediated inflammation and CIR mutually reinforce each other, ultimately impairing neuronal bioenergetics via transcriptional repression of glycolytic machinery (5, 24, 25). Preclinical evidence from the diabetic mouse model reveals this reciprocal relationship, demonstrating significant upregulation of pro-inflammatory M1 marker concurrent with downregulation of neuroprotective M2 marker, findings that correlate strongly with observed cognitive impairments (26). Furthermore, in db/db mice, the hyperglycemic environment of diabetes stimulates microglia to produce reactive oxygen species and activates the NF-κB/NLRP3 signaling pathway, leading to the production of NLRP3 inflammasome (e.g., IL1β, IL6, and IL18), which in turn mediates cognitive dysfunction (27). Oxidative stress is a major pathogenic culprit that leads to metabolic anomalies, as well as neurodegeneration and aging. Accumulating preclinical and clinical studies suggest that oxidative stress contributes to neuronal loss and synaptic disruption through impairment of mitochondrial homeostasis in the brain, ultimately resulting in cognitive dysfunction (28, 29).

Omega-3 PUFAs, particularly DHA and EPA, exhibit multifaceted neuroprotective effects in DACD by modulating interconnected inflammatory and oxidative pathways. First, omega-3 PUFAs mitigate chronic low-grade neuroinflammation associated with hyperglycemia by inhibiting the release of proinflammatory cytokines (e.g., IL-1β, TNF-α) and NF-κB inflammatory signaling pathway transduction (30, 31). Second, they inhibit inflammasome activation (e.g., NLRP3) by reducing oxidative stress and mitochondrial dysfunction, critical drivers of neuronal damage in diabetes (32). Third, omega-3 PUFAs regulate microglia-mediated synaptic pruning and plasticity by suppressing 12/15-lipoxygenase (LOX)/12-HETE signaling, thereby preventing excessive phagocytosis of synaptic elements during neurodevelopment (33). Additionally, omega-3 PUFAs are indispensable components of cell membranes and play an important role in maintaining the membrane structural integrity and fluidity of immune and neuronal cells (31). Moreover, DHA integrates into neuronal membranes, stabilizing lipid rafts and suppressing microglial overactivation triggered by advanced glycation end products (34). Finally, omega-3 PUFAs, particularly EPA, mitigate DACD by suppressing oxidative stress through activation of the P62/KEAP1/NRF2 antioxidant pathway, which reduces reactive oxygen species generation and subsequent neuronal damage (35). In conclusion, omega-3 PUFAs represent a promising dietary intervention targeting the neuroinflammatory-oxidative axis in DACD.

## 4 Omega-3 PUFAs: synaptic architects in diabetic cognitive protection

Emerging as a neural epicenter of DACD, the hippocampus exhibits signature neurodegenerative changes—particularly synaptic diminution and dendritic retraction-that correlate strongly with clinical disease progression. The function of the hippocampus depends on communication among neurons, and the synapse is the basic information-processing unit that mediates neuronal communication. Preclinical investigations in diabetic models reveal marked synaptic alterations, characterized by diminished spine density, downregulation of synaptic scaffolding proteins (postsynaptic density protein-95, microtubule-associated protein 2), and ultrastructural abnormalities including truncated postsynaptic densities and expanded synaptic clefts (36, 37). These pathological changes correlate strongly with cognitive deficits observed in db/db mice. Omega-3 PUFAs, notably DHA which comprises ~30% of brain phospholipid content, exhibit significant neuroprotective effects at synaptic sites. Experimental studies demonstrate their ability to enhance long-term potentiation, maintain synaptic ultrastructural integrity, and increase expression of activity-regulated cytoskeleton-associated protein, a critical molecular mediator of synaptic plasticity and memory consolidation processes (38, 39). Phosphatidylserine is predominantly localized on the cytoplasmic side of neuronal cell membranes, in facilitating the action of signaling proteins that underpin neuronal survival, neurite growth, and synaptogenesis. DHA orchestrates neural membrane homeostasis through dual actions-stimulating phosphatidylserine-dependent signaling hubs for neurodevelopment while structurally optimizing bilayer fluidity for synaptic transmission efficiency (40). This bifunctional capacity underlies its essential role in neuronal circuit formation and function.

Transcending their membrane-stabilizing functions, omega-3 PUFAs emerge as master regulators of neurotrophic signaling networks, orchestrating complex interactions between growth factor systems and synaptic efficacy pathways. Dietary supplementation with DHA enhances brain-derived neurotrophic factor (BDNF) expression via cAMP response element-binding protein-dependent transcriptional activation, thereby promoting synaptogenesis and supporting cognitive processes (41, 42). DHA-derived neuroprotectin D1 provides additional protection by balancing Bcl-2/Bax ratios to inhibit apoptosis and promote autophagy of damaged organelles. DHA can also clear damaged mitochondria and alleviate mitochondrial dysfunction through PINK1/Parkin mediated mitophagy and also increases acetylcholine and γ-aminobutyric acid levels while decreasing glutamate levels, ultimately counteracting the synaptotoxic effects of diabetes (43, 44). Additionally, omega-3 PUFAs as master regulators of a gut-brain circuit, stimulating short-chain fatty acids (SCFA) production that bridges microbial metabolism to neuronal plasticity. Notably, these synaptic alterations may be further modulated by gut-derived inflammatory signals, as discussed in the following gut-brain axis section.

## 5 Omega-3 PUFAs: guardians of the gut-brain axis in diabetes

The gut-brain axis represents a sophisticated bidirectional communication network linking gut microbiota with brain function through immune, neuroendocrine, and metabolic pathways. Diabetes-associated gut dysbiosis orchestrates a dual-hit mechanism: SCFAs depletion undermines epithelial integrity while lipopolysaccharide and toll-like receptor 4 engagement activate pro-inflammatory NF-κB cascades, establishing a systemic-to-neural inflammatory axis (45, 46). Dysbiosis of gut microbiota can also induce deficits in synaptic plasticity through the ER stress-mediated PERK signaling pathway (47). These pathological changes correlate strongly with the microbial alterations and intestinal damage observed in diabetic patients. Omega-3 PUFAs, especially EPA and DHA, emerge as potent modulators of this axis. Omega-3 PUFAs modulate gut microbiota diversity, enriching beneficial taxa (e.g., *Bifidobacterium, Lactobacillus*) and increasing SCFAs production, which enhances intestinal barrier integrity (11, 48). SCFAs, particularly butyrate, cross the BBB to suppress microglial activation and upregulate BDNF expression. Omega-3 PUFAs and DHA also up-regulated expression of the intestinal tight junction protein occludin and zonula occluden-1, improved the intestinal barrier functions and repaired BBB damage (44). Moreover, omega-3-derived endocannabinoids interact with gut vagal afferents to regulate appetite and glucose homeostasis, creating a holistic approach to metabolic and cognitive protection in diabetes (46, 49).

## 6 Discussion with future perspectives

**Figure 1:**
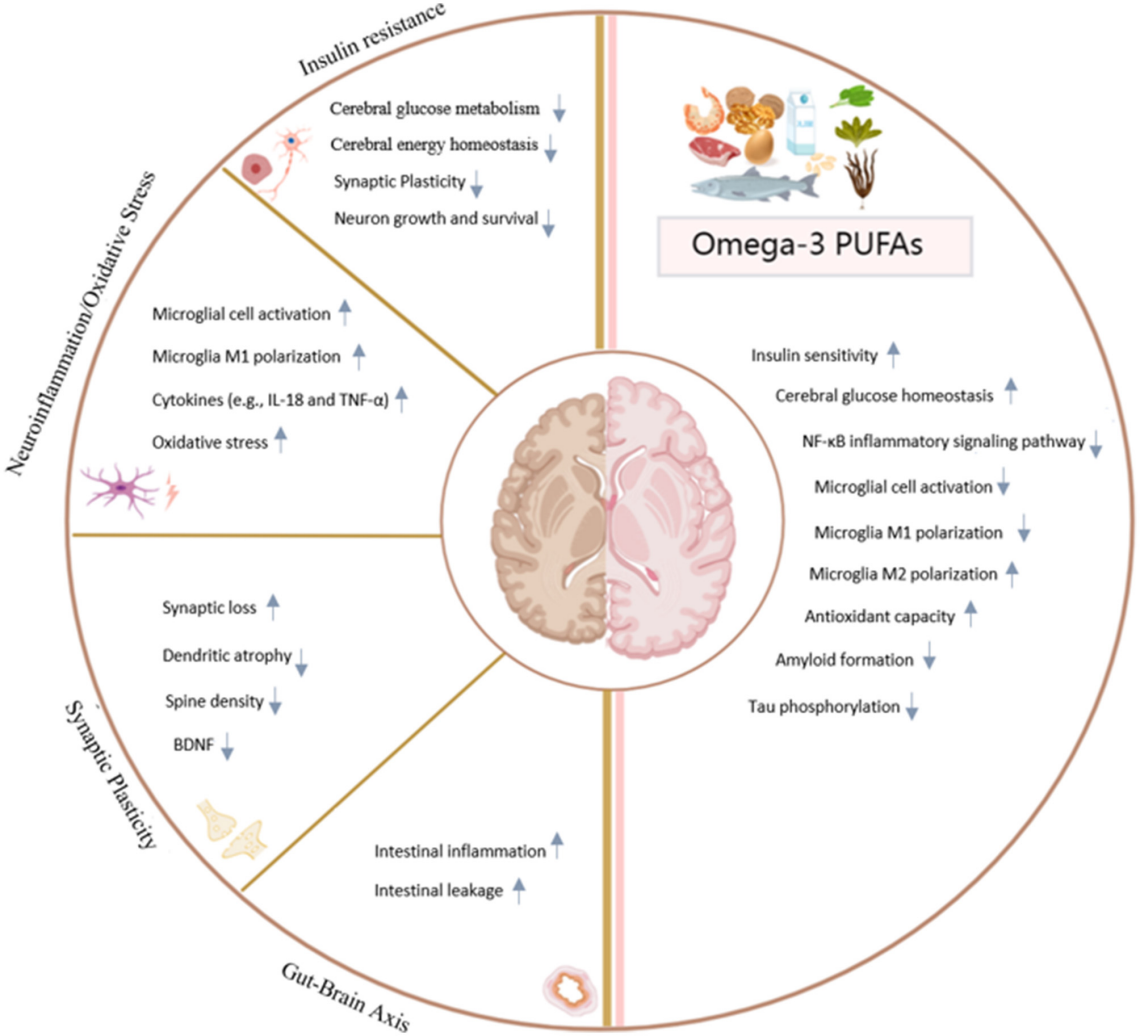
Regulatory mechanism of omega-3 PUFAs on diabetic-associated cognitive dysfunction. Created in MedPerr.com.

Omega-3 PUFAs, especially DHA and EPA, exhibit pleiotropic therapeutic effects against DACD through multiple complementary mechanisms (Figure 1). These essential fatty acids (i) restore cerebral insulin sensitivity via IRS/PI3K/AKT signaling pathway activation, (ii) attenuate neuroinflammation by promoting PPARγ-mediated microglial polarization toward the neuroprotective M2 phenotype, and (iii) enhance synaptic plasticity through BDNF upregulation. Furthermore, their modulation of the gut-brain axis—marked by elevated butyrate production and SCFAs-dependent strengthening of the blood-brain barrier-contributes to systemic anti-inflammatory effects. At the molecular level, DHA demonstrates amyloid-lowering properties while EPA-derived specialized pro-resolving mediators actively resolve inflammation, synergistically protecting hippocampal integrity.

Current evidence suggests that diabetic patients may benefit from regular consumption of omega-3 PUFAs-rich foods, though the optimal frequency and dose still need to be confirmed. Although plant-based omega-3 PUFAs have limited capacity to convert into bioactive EPA/DHA, supplementation with marine-derived omega-3 PUFAs shows preliminary promise, particularly for neuroprotection with higher DHA proportions, but ideal ratios remain under investigation. Genetic factors (e.g., APOE ε4) may influence dosing needs, and monitoring tools such as the omega-3 PUFAs index are still in the exploratory phase, awaiting the establishment of reliable thresholds based on randomized controlled trials. Therefore, recommended protocols should be personalized based on emerging evidence and clinical context.

While our findings demonstrate the therapeutic potential of omega-3 PUFAs for DACD, several key challenges must be addressed in future clinical translation. First, the optimal EPA:DHA ratio and dosage regimen remain to be established, particularly considering the metabolic heterogeneity of T2DM populations and potential pharmacodynamic interactions with glucose-lowering medications. Second, standardized cognitive assessment protocols sensitive to early DACD progression are urgently needed, as current diagnostic criteria lack specificity for diabetes-related cognitive decline. Third, the development of validated biomarkers—including erythrocyte omega-3 PUFAs indices, neuroimaging parameters, and inflammatory markers—will be critical for demonstrating target engagement and dose-response relationships in clinical trials. Finally, genetic variations (e.g., APOE ε4), baseline nutritional status, comorbidities, and concomitant medication use must be considered to achieve personalized treatment in different populations. Addressing these challenges through multidisciplinary collaborations will accelerate the development of evidence-based omega-3 PUFAs interventions for DACD prevention and management.
